# A rare case of single right coronary artery with congenital absence of left coronary artery in an adult: a case report

**DOI:** 10.1186/s13019-015-0267-0

**Published:** 2015-04-21

**Authors:** Fengli Fu, Hongfeng Jin, Yue Feng

**Affiliations:** 1 Department of Radiology, Zhejiang Hospital, 12 Lingyin Road, Hangzhou, 310013 People's Republic of China; 2 Department of Cardiology, Zhejiang Hospital, 12 Lingyin Road, Hangzhou, 310013 People's Republic of China

**Keywords:** Coronary anomalies, Single right coronary artery, Computed tomography

## Abstract

Single right coronary artery with congenital absence of left coronary artery is one of the rarest coronary artery anomalies. Most coronary anomalies are asymptomatic and incidental findings. We report a case of single right coronary artery with congenital absence of left coronary artery detected by coronary CT angiography. Physical examination revealed a well-nourished female with a blood pressure of 130/75 mmHg and a pulse rate of 56 beats per minute. The myocardial enzymes and blood lipid levels showed normal findings. The dynamic electrocardiogram revealed frequent ventricular premature beats. Dual-source CT angiography was performed for evaluation of coronary artery. The imaging showed a very large single coronary artery arising from the right coronary sinus of Valsalva, and demonstrated absence of the left coronary artery. Meanwhile, the findings were confirmed by coronary angiography.

## Background

A single coronary artery (SCA) is a rare congenital anomaly of the coronary arteries, which is described as one coronary artery arising from the aortic trunk by a single coronary ostium and providing for perfusion of the entire myocardium [1-3]. The prevalence of SCA is approximately 0.024% [1] to 0.066% [2] in population who undergo coronary angiography.

In the last century, different classification systems for SCA based on necropsy findings and angiographic variants were suggested [4]. According to the site of origin and anatomical distribution of the branches, Lipton et al. [1] proposed a very useful angiographic classification. Based on the classification, the primary division was made between the ‘R’ right-type and ‘L’ left-type according to the site of origin of SCA in the right or left sinus of Valsalva. Furthermore, each case was designated as belonging to group I, II or III depending on the anatomical course of the artery. Single right coronary artery with congenital absence of left coronary artery is extremely rare, and very few cases have been reported. In the following case, we describe a single right coronary artery with congenital absence of left coronary artery detected by coronary CT angiography (CTA).

## Case presentation

**Figure 1:**
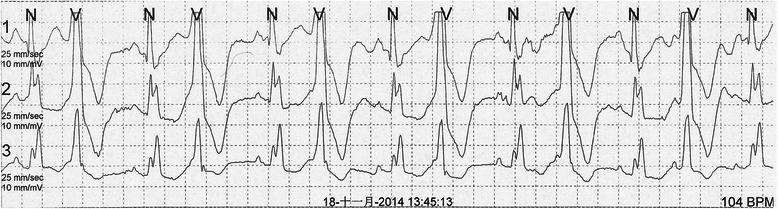
Electrocardiogram. Dynamic electrocardiogram revealed frequent ventricular premature beats.

A 63-year-old Chinese woman was referred to our hospital for unprovoked oppression in chest for one month, without palpitation, syncope, and nocturnal dyspnea. The symptom lasted tens of minutes and recovered automatically after rest. Physical examination revealed a well-nourished female with a blood pressure of 130/75 mmHg and a pulse rate of 56 beats per minute. Cardiac auscultation revealed premature beat. The myocardial enzymes and blood lipid levels showed normal findings. The dynamic electrocardiogram revealed frequent ventricular premature beats (Figure 1). Her clinical condition was suspected to be caused by coronary arterial disease, so she was further investigated by coronary CTA.

**Figure 2:**
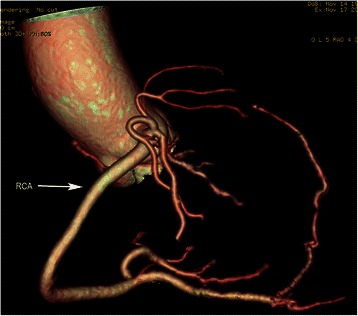
Volume-rendered coronary tree. A single right coronary artery (RCA) arises from the right coronary sinus of Valsalva, and shows absence of the left coronary artery.

**Figure 3:**
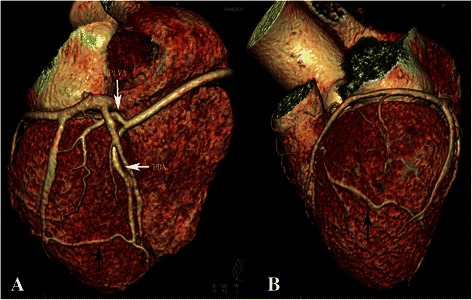
Volume-rendered reconstruction. **(A)** After branching off posterior left ventricular artery (PLVA), the posterior descending artery (PDA) gives off a small branch (black arrow) in rear of cardiac apex. **(B)** This small branch (black arrow) terminates at anterior wall of the left ventricular.

The examination was performed on a dual-source CT (Somatom Definition, Siemens Medical Systems, Forchheim, Germany). Coronary angiography scan was administered by continuously injecting a bolus of 70 ml of iopromide (Ultravist 370, 370 mg I/ml, Bayer Healthcare, Berlin, Germany) followed by 50 ml saline solution via a power injector into an antecubital vein (at a rate of 4.5 mL/s). 3D volume rendering image showed a very large single coronary artery arising from the right coronary sinus of Valsalva, and demonstrated absence of the left coronary artery (Figure 2). The single right coronary artery (RCA) coursed the anatomic position of the normal right coronary artery. The perfusion of anterior interventricular septum was provided by one branch arising from the initial part of RCA. After branching off posterior left ventricular artery (PLVA), the RCA continued through as a posterior descending artery (PDA), giving off a small branch in rear of cardiac apex to supply the lateral wall of the left ventricle (Figure 3). An aberrant-coursing artery with small diameter originated from the distal of posterior lateral ventricular artery to supply the lateral wall of the left ventricle. CTA revealed the lumen of the initial PDA was mild narrowed by atherosclerotic plaques. No calcifications were detected in coronary artery walls, and the calcium score was zero.

**Figure 4:**
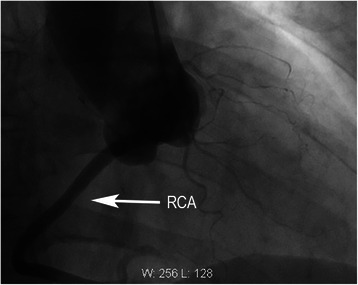
Coronary angiography. A single right coronary artery (RCA) arises from the right coronary sinus of Valsalva.

**Figure 5:**
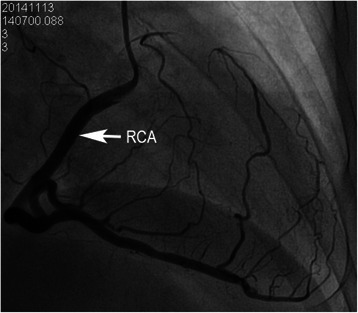
Coronary angiography. The entire coronary arterial circulation is supplied by a single right coronary artery (RCA) arising from the right sinus of Valsalva.

The diagnosis that there is a single right coronary artery with absence of left coronary artery, as well as atherosclerotic plaques of the posterior descending artery, was confirmed by coronary angiography (Figures 4, 5). When making the diagnosis of this case report, the differential diagnosis, such as occlusion of the left main coronary artery and left coronary artery should be considered, which usually presents one funicular in course of coronary artery. No abnormity was showed in systolic and diastolic function of the left ventricular in left ventricular angiography. Finally, the patient accepted electrophysiology test and radiofrequency ablation to treat ventricular premature beats.

## Conclusions

The coronary arterial circulation may rarely be supplied by a single coronary artery (SCA) arising from either the right, left or posterior sinus of Valsalva [5]. An isolated single right coronary artery anomaly was defined as R-I type, based on the classification proposed by Lipton et al. and further modified by Yamanaka and Hobbs [1,3]. It is one of the rarest coronary artery anomalies of SCA.

According to our search of the literature, fewer than 4 cases of R-I type had been reported up to 2014. In the previously studies [1,3,6], the single RCA followed the course of normal RCA and continued as a left circumflex artery (LCA) in the left atrioventricular groove, extending as the left anterior descending artery (LAD). However, in our case, the distal of PDA gives off a small branch in rear of cardiac apex, and terminates at anterior wall of the left ventricular. An aberrant-coursing artery with small diameter originated from the distal of PLVA. The left ventricular was supplied by three branch of RCA. It may be a new subtype of the Lipton R-I.

The etiology of SCA is uncertain. In some cases, SCA may lead to life threatening symptoms, including angina pectoris, myocardial infarction, syncope, cardiac arrhythmias, congestive heart failure, or sudden death [1]. Despite no syndrome was found after radiofrequency ablation and the hemodynamic status of single right coronary artery showed benign in the present case, we encourage close follow-up, because obstruction of a SCA could be fatal.

Coronary CTA provides accurate angiographic information on the origin, course, and termination of coronary anomalies non-invasively. Three-dimensional reconstruction is useful for visualizing this condition. Diagnostic key point of the single right coronary artery with congenital absence of left coronary artery is that the coronary arterial circulation was supplied by a single right coronary artery arising from the right sinus of Valsalva. Having a knowledge of this rare anomaly will be helpful in the differential diagnosis of coronary artery abnormalities and assist the physician in treatment planning.

## Consent

Written informed consent was obtained from the patient for publication of this case report and any accompanying images. A copy of the written consent is available for review by the Editor-in-Chief of this journal.

## Abbreviations

CTA: CT angiography
RCA: Right coronary artery
PLVA: Posterior left ventricular artery
PDA: Posterior descending artery
SCA: Single coronary artery
LCA: Left circumfex artery
LAD: Left anterior descending artery
